# Growth trajectories in the cave bear and its extant relatives: an examination of ontogenetic patterns in phylogeny

**DOI:** 10.1186/s12862-015-0521-z

**Published:** 2015-11-02

**Authors:** Manuela Fuchs, Madeleine Geiger, Madlen Stange, Marcelo R. Sánchez-Villagra

**Affiliations:** Palaeontological Institute and Museum, University of Zurich, Karl-Schmid-Strasse 4, Zürich, 8006 Switzerland

**Keywords:** Mammalia, *Ursus*, Ontogeny, Variation, Disparity, Evolution, Morphometrics, Cranium, Mandible

## Abstract

**Background:**

The study of postnatal ontogeny can provide insights into evolution by offering an understanding of how growth trajectories have evolved resulting in adult morphological disparity. The *Ursus* lineage is a good subject for studying cranial and mandibular shape and size variation in relation to postnatal ontogeny and phylogeny because it is at the same time not diverse but the species exhibit different feeding ecologies. Cranial and mandibular shapes of *Ursus arctos* (brown bear), *U. maritimus* (polar bear), *U. americanus* (American black bear), and the extinct *U. spelaeus* (cave bear) were examined, using a three-dimensional geometric morphometric approach. Additionally, ontogenetic series of crania and mandibles of *U. arctos* and *U. spelaeus* ranging from newborns to senile age were sampled.

**Results:**

The distribution of specimens in morphospace allowed to distinguish species and age classes and the ontogenetic trajectories *U. arctos* and *U. spelaeus* were found to be more similar than expected by chance. Cranial shape changes during ontogeny are largely size related whereas the evolution of cranial shape disparity in this clade appears to be more influenced by dietary adaptation than by size and phylogeny. The different feeding ecologies are reflected in different cranial and mandibular shapes among species.

**Conclusions:**

The cranial and mandibular shape disparity in the *Ursus* lineage appears to be more influenced by adaptation to diet than by size or phylogeny. In contrast, the cranial and mandibular shape changes during postnatal ontogeny in *U. arctos* and *U. spelaeus* are probably largely size related. The patterns of morphospace occupation of the cranium and the mandible in adults and through ontogeny are different.

**Electronic supplementary material:**

The online version of this article (doi:10.1186/s12862-015-0521-z) contains supplementary material, which is available to authorized users.

## Background

Understanding the evolution of skull shape by examining skull growth trajectories of related species helps to understand the modifications of cranial structures that arise in relation to diet, size, and phylogeny. Growth trajectories of skull shape have been studied in diverse vertebrate groups such as in, among many other taxa, *Triturus* newts [1], *Podarcis* lizards [2], chelid turtles [3], *Caiman* species [4], avian and non-avian dinosaurs [5], the rodents *Sigmodon fulviventer* [6] and *Thrichomys apereoides* [7], the spotted hyena (*Crocuta crocuta*) [8], the felids *Puma concolor* [9], *Herpailurus yagouaroundi*, and *Acinonyx jubatus* [10], the canid *Lycalopex culpaeus* [11], the common and pigmy hypopotami *Hippopotamus amphibious* and *Hexaprotodon liberiensis* [12], the apes *Pan paniscus* and *Pan troglodytes* [13], Neanderthals [14], and modern humans [15]. Findings in canids are that size and shape stand in relation to dietary shift after weaning [11]; in hyenids, skull size and shape maturity precedes sexual maturity due to strong competition for food [8]; in felids, ontogenetic shape change is due to size and is not constrained by phylogeny [10]. All in all, investigated clades seem to express different modes of skull shape trajectories and different variables that affect them, without a single, universal pattern and mechanism behind them. More studies, including also extinct taxa are needed to further document and understand the diversity of patterns of cranial shape change during ontogeny and across evolutionary time scales.

**Fig. 1:**
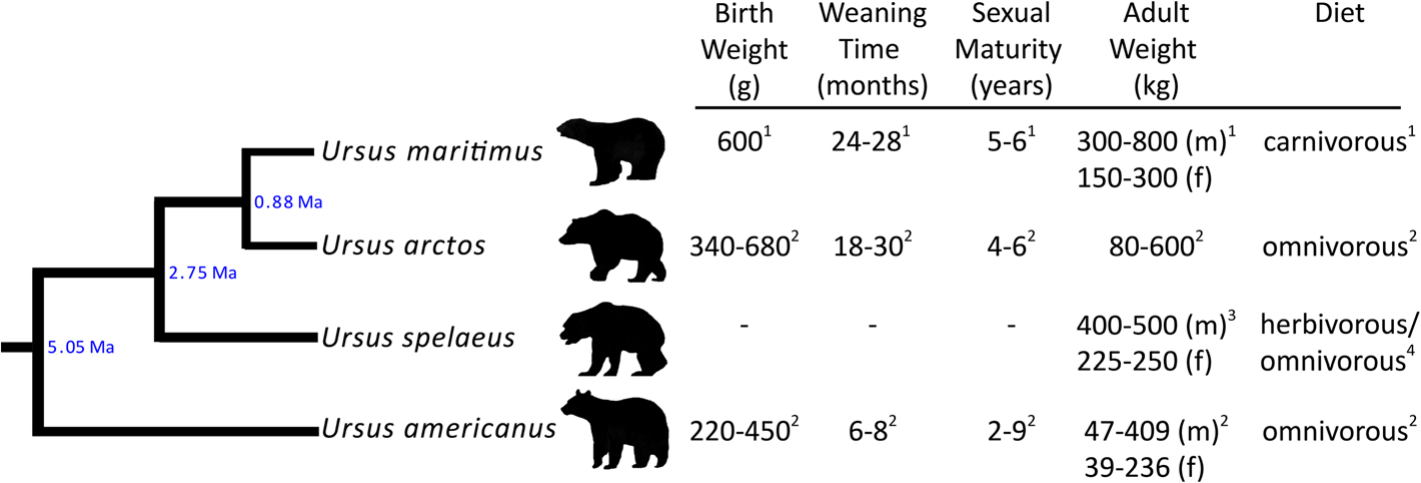
Phylogenetic tree and life history data of the investigated species. The divergence times are based on molecular data suggested by Krause et al. [16]. Life history data were taken from the literature: 1, De Maseter & Stirling [56]; 2, Myers et al. [49]; 3, Christiansen [57]; 4, Bocherens et al. [58], Hilderbrand et al. [52], Stiner et al. [59], Richards et al. [51], Figueirido et al. [29]. m, male; f, female

Geometric morphometric (GM) studies on ontogenetic trajectories were already conducted on diverse groups of carnivorans. In this study, three-dimensional (3D) GM are applied for the first time on species of the genus *Ursus*, a lineage that is not particularly diverse and contains herbivorous, omnivorous, and carnivorous species. This clearly constitutes an advantage of the present study, as shape change among relatively few closely related species and these feeding ecologies can be investigated. The extant *Ursus* lineage includes *U. arctos* (brown bear), *U. maritimus* (polar bear), *U. americanus* (American black bear), and *U. thibetanus* (Asiatic black bear). Several aspects of the timing and patterns of divergence of ursid species have been investigated in recent years [16–24] (Fig. 1). In this study, we investigate intra- and interspecific cranial shape changes in extant *U. arctos*, *U. maritimus*, and *U. americanus* and compare the shape changes and life history traits to the extinct relative, *U. spelaeus* (cave bear), in order to trace and understand the evolutionary skull shape change. Because different diets demand specific adaptations of the jaw musculature and skull shape, we expect to detect shape modifications associated with dietary changes among adults of different species and changes from sub-adult period to adulthood. Life-history information of *U. spelaeus* and of the extant species examined is listed in Fig. 1. Although fossils harbour the difficulties of incomplete and insufficient sampling, it is worth to include them into a study of skull growth trajectories, since they, even if fragmentary, shed light on the evolution of observed changes and the generation of extant phenotypic disparity

*U. spelaeus* appeared around 126,000 years before present, at the beginning of the last interglacial [25] and became extinct during the last glacial-interglacial cycle around 27,800 years before present [26]. Innumerable remains of *U. spelaeus* have been found in caves throughout Europe, which have accumulated over a period of thousands of years. Not only the quantity of the remains, but also their well-preserved condition makes this species a particularly valuable object for paleontological research. It provides a unique possibility to examine the morphological variation [27], feeding ecology [28, 29], and ontogeny [30, 31] of this species together with its extant relatives. Previous studies about cranial shape variation in *U. spelaeus* focused on intraspecific variation [27] and ecomorphology [28, 29, 32, 33]. A strong correlation between feeding ecology and craniodental morphology in the Ursidae lineage was reported by Sacco & Van Valkenburgh [34] and Christiansen [28]. However, there are only few studies on patterns of ontogeny of *U. spelaeus* crania in comparison to extant relatives. In a detailed study, Ehrenberg [30] described and compared a neonate *U. spelaeus* specimen with a *U. arctos* neonate as well as the ontogeny of these two species. Another work focused on dental eruption in *U. spelaeus* and *U. arctos* [35].

In this study, 3D GM and multivariate analyses are used to assess and compare the extent of overlap or dissociation of extinct (*U. spelaeus*) and extant bear (*U. arctos*, *U. maritimus*, *U. americanus*) skull shapes in morphospace. The cranial and mandibular growth of the *Ursus* lineage is approached in a comparative perspective. The following questions are addressed: a) how does adult skull and mandibular shape vary among the bear species? b) are skull and mandibular shape constrained by phylogeny? c) are intra- and interspecific skull and mandibular shape variation and disparity driven by size differences? e) how does skull and mandibular shape change during ontogeny? f) are ontogenetic trajectories conserved during evolution?

## Methods

### Specimens

A total of 253 crania and 183 mandibles of the extinct *U. spelaeus* and the extant species *U. arctos*, *U. maritimus*, and *U. americanus*, were investigated (Table 1). The sample consists of adults of both sexes. Juvenile specimens were included for *U. arctos* and *U. spelaeus. U. arctos* was chosen for comparison of ontogenetic trajectories with *U. spelaeus* due to its close phylogenetic relationship to *U. spelaeus*, its similar diet and area of distribution, and good availability of material (Fig. 1). None of the specimens appeared to have exhibited pathologies that affected skull shape.

**Table 1:** Number (n) of specimens examined in this study

Species	n (cranium)			n (mandible)		
	Adults	Juveniles	Total	Adults	Juveniles	Total
*U. arctos*	56	27	83	56	21	77
*U. americanus*	28	-	28	28	-	28
*U. maritimus*	42	-	42	43	-	43
*U. spelaeus*	97	3	100	31	4	35

The investigated crania and mandibles are housed in the collections of several institutions in Europe: **IfPEN**, Institut für Paläontologie Erlangen, Erlangen, Germany; **IPUW**, Institut für Paläontologie Wien, Vienna, Austria; **MCP**, Château de Montbéliard, Montbéliard, France; **NHMW**, Naturhistorisches Museum Wien, Vienna, Austria; **NMB**, Naturhistorisches Museum Basel, Basel, Switzerland; **NMBE**, Naturhistorisches Museum Bern, Berne, Switzerland; **NMSG**, Naturmuseum St. Gallen, St. Gallen, Switzerland; **RBIN**, Institut Royal des Sciences Naturelles de Belgique, Brussels, Belgium; **ZMUZH**, Zoologisches Museum der Universität Zürich, Zurich, Switzerland. The specimens of *U. spelaeus* have been found in various caves in Central Europe and the Middle East: Gondenans-les-Moulins, Goyet Cave, Zoolithenhöhle, Mixnitz, Salzhofenhöhle, Sloupa, Kiriteinenhöhle, Conturines, Ramesch, Schwabenreith. More detailed information on the sample can be found in Additional file 1: Table A1. Unless not stated otherwise, all analyses were performed using MorphoJ 1.06b, an integrated software package for geometric morphometrics [36].

### Age and sex determination

The examined specimens were classified as juvenile, corresponding to the individual dental age stages IDAS 1 and 2 (permanent dentition not yet completed), or adult, corresponding to IDAS 3 to 5 (completed eruption of permanent dentition into occlusion) [37]. Sexual dimorphism in the *Ursus* lineage is primarily expressed through a larger body size of adult males compared to adult females as well as through differences in total size of the canine teeth [28, 38–40]. No sexual dimorphism is generally apparent in juveniles [31, 40]. Consistent sexing of *U. speleaus* and extant bear species of unknown sex according to dental measurements described by Gordon & Morejohn [40] was not feasible because of great adult size variation among specimens from different populations. This issue has already been noted by Gordon & Morejohn [40]. Therefore, shape and size differences based on sexual dimorphism were not considered in this study.

### Landmarks and data analysis

**Fig. 2:**
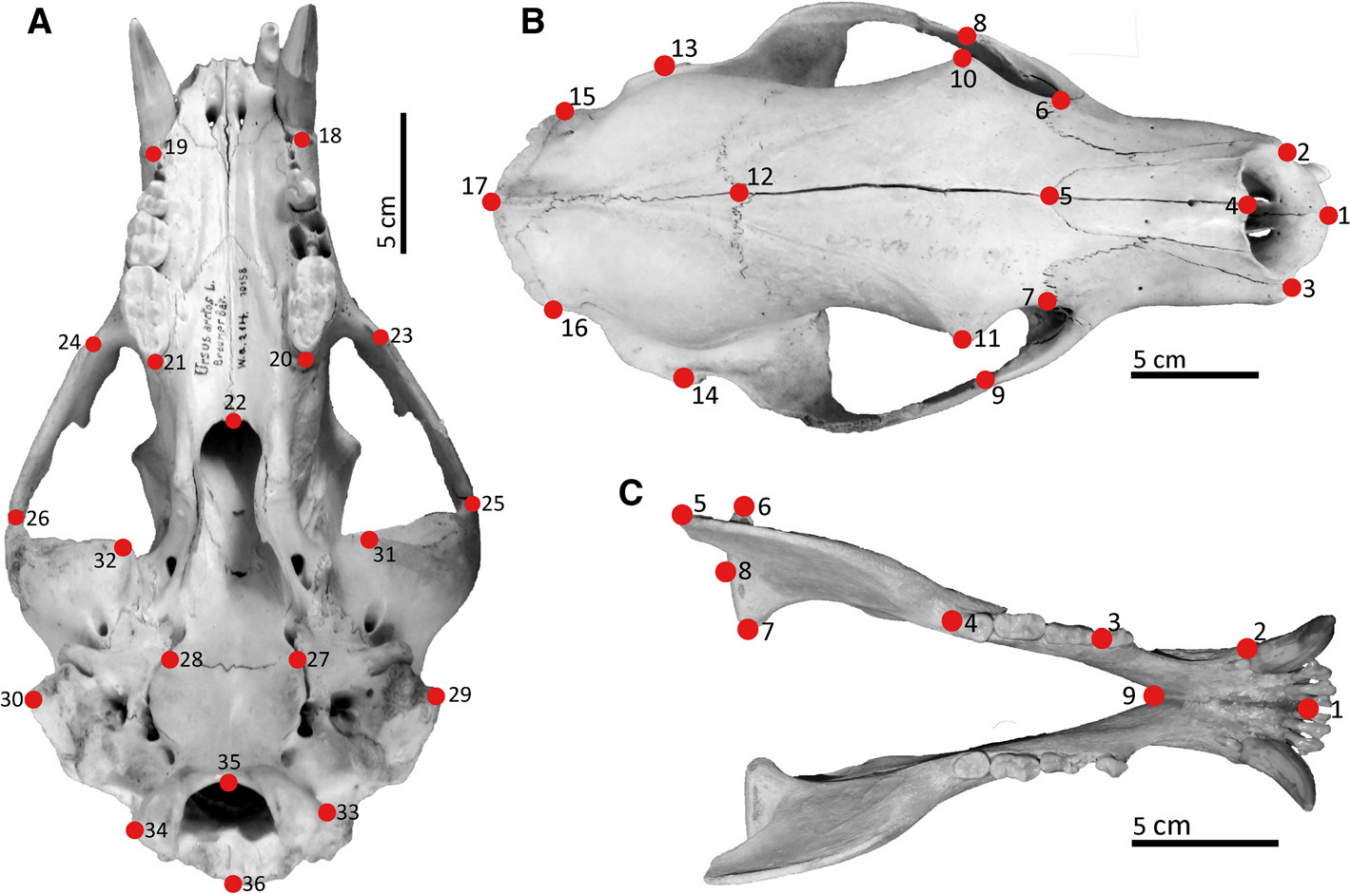
Cranial landmarks used in the morphometric analysis. Landmarks are illustrated on *U. arctos* ZMUZH10158 in ventral (**a**) and dorsal (**b**) aspects. Mandibular landmark positions illustrated on *U. maritimus* BA3270 in dorsal aspect (**c**)

Thirty-six landmarks describe both lateral sides of the cranium and nine landmarks describe one side of the mandible (Table 2, Fig. 2). The landmarks represent homologous structures that are clearly recognisable in every age stage and in all four species examined. To digitize the skulls in three dimensions, a MicroScribe MX 3D digitizer (Solution Technologies, Inc.) with 5° of freedom and an accuracy of 0.1016 mm was used.

**Table 2:** List of cranial and mandibular landmarks and their definition used in this study (Fig. 2)

Cranial landmarks (dorsal)	
1	Anterior point of the interpremaxillary suture at the alveolar margin of the incisors
2/3	Anterior point of the premaxillo-maxillary suture at the alveolar margin of the incisors
4	Anterior point of the internasal suture
5	Intersection of internasal and interfrontal sutures
6/7	Dorsal point of the lacrimal bone where it meets the frontal bone and the maxilla
8/9	Dorsal tip of the frontal process at the zygomatic arch
10/11	Tip of the post-orbital process
12	Intersection of the interparietal and interfrontal sutures
13/14	Posterior point of the external auditory meatus
15/16	Intersection of parietal, squamosal, and supraoccipital bones
17	Distal point of the external occipital protuberance
Cranial landmarks (ventral)	
18/19	Posterior point of the canine alveolus
20/21	Posterior point of the tooth row at the alveolar margin
22	Posterior point of maximum concavity on the palatine
23/24	Ventral point of the jugo-maxillary suture
25/26	Ventral point of the jugo-squamosal suture
27/28	Intersection of basioccipital, basisphenoid, and auditory bulla
29/30	Tip of the mastoid process
31/32	Ventral tip of the postglenoid process
33/34	Lateral point of the occipital condyle
35	Antero-ventral point of the foramen magnum
36	Postero-dorsal point of the foramen magnum
Mandibular landmarks	
1	Antero-ventral point of the mandibular symphysis and anterior part of the alveolar margin of the incisor
2	Postero-dorsal border of the canine alveolus
3	Anterior point of the alveolar margin of p4
4	Posterior point of the alveolar margin of the tooth row
5	Posterior edge of the coronoid process
6	Lateral edge of the articular surface of the condyloid process
7	Medial edge of the articular surface of the condyloid process
8	Tip of the angular process
9	Ventral point of the symphyseal region

In some fossil *U. spelaeus* specimens certain cranial landmarks are missing. These missing landmarks were extrapolated with the R-function “estimate.missing” implemented in the R package “geomorph” v.2.1 [41]. The landmark data of all crania and mandibles were superimposed using Generalized Procrustes analysis [42–44]. This method optimally translates and rotates the homologous landmarks and scales them to the unit centroid size to minimize the difference between landmark configurations, which makes it easier to compare the shape of different objects. Because both sides of the cranium were used, only the symmetric components of the averaged (left and right sides of the skull) landmarks were used for calculating a covariance matrix and subsequent multivariate analyses. A principal component analysis (PCA) including all four species was performed. The PCA identifies patterns of variation and covariation of the landmark configurations and simplifies them by replacing the original variables with new ones (principal components, PCs) representing major axes of variance. PCs are linear combinations of the original variables and independent of each other [45]. Shape changes along the *Ursus* lineage can thus be visualized in a morphospace, investigating the position of taxa along those major axes of variance. The PCA was used to study the distribution of the specimens considering different species and age classes in morphospace.

### Cranial shape changes in relation to size

To investigate the extent to which the shape variation is associated with size, regression analyses of the Procrustes coordinates onto log centroid size were performed. Centroid size, used as an estimate for skull size, is defined as the squared root of the sum of squared distances of each landmark from the centroid of the landmark configuration [45, 46]. In this way the effect of allometry was eliminated. PCA were then conducted on the regression residuals. For comparison of the size associated shape modifications of *U. arctos* and *U. spelaeus*, the angles between the corresponding regression vectors were calculated and similarity between them was tested as described by Drake & Klingenberg [47].

### Age stage and species differentiation

To calculate Procrustes distances between species and age stages, canonical variates analyses were conducted for cranial and mandibular landmarks, using ontogenetic stages (two groups) and species (four groups) as classifier variables. Procrustes distances were calculated based on Procrustes coordinates and on regression residuals of Procrustes coordinates (to remove the effect of allometry among species), respectively. The significance of the group differences was tested with permutation tests using 10,000 resamples.

### Phylogenetic comparison

By mapping known phylogenetic trees onto size corrected PC scores [16], the effect of phylogeny on shape changes was investigated. In other words, we investigated whether similarity in shape correlates with phylogenetic relatedness. The phylogenetic signal was tested simulating the null hypothesis of the complete absence of a phylogenetic signal by randomly permuting the phenotypic data using 10,000 iterations [48]. For this purpose, the total amount of squared change, summed over all branches of the tree was used. The analysis was performed once based on the divergence times suggested by Krause et al. [16] and once using a phylogenetic tree with branch lengths set as equal to one for all taxa. Only PC scores of adult specimens were included in this analysis and branches were not weighted.

## Results

### Shape variation in cranial morphology

**Fig. 3:**
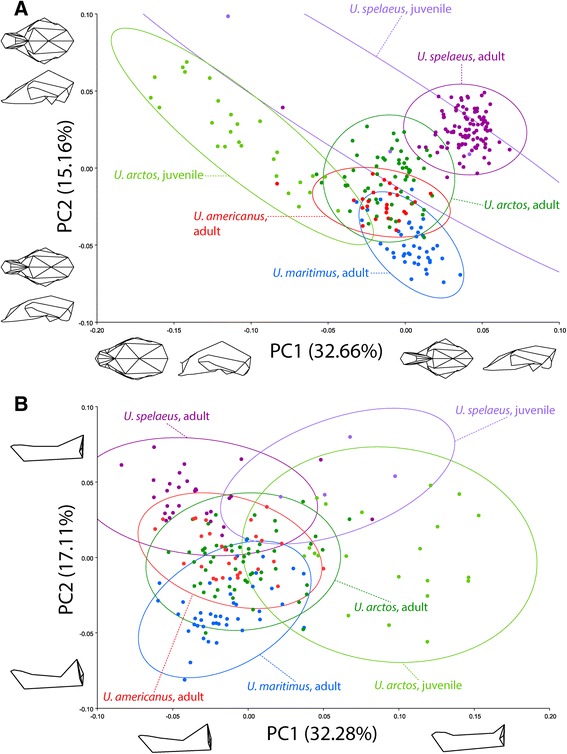
Principal component analysis of cranial (**a**) and mandibular (**b**) shape variation in juvenile and adult bear species. Ellipses represent the 95 % confidence interval of the species and age stages. Cranial and mandibular models represent extreme shape on PC1 and PC2

The first three principal components of cranial shape across species account for 56.14 % of the total variation. PC1 is associated with a set of transformations that separates the juvenile *U. arctos* and *U. spelaeus* from adult *U. spelaeus*; the adults from the extant species are centered on PC1 (Fig. 3a & Additional file 2: Figure A1). Juvenile specimens are characterised by a relatively short and wide face and palate as well as a rounded skull with a high and domed braincase (Fig. 3a). In addition, the zygomatic arches are relatively contracted and the foramen magnum is ventrally oriented. Along PC1, from negative to positive values, the whole cranium elongates, the braincase flattens, and the zygomatic arches expand. Especially the rostrum gets relatively long and narrow. The foramen magnum shifts to a more posterior position and the prebasial angle is more declined (ventrally rotated) (Fig. 3a). The juvenile *U. arctos* and *U. spelaeus* are separated from their adult conspecific on PCs 1 and 2 in an approximately 45° angle to the adults (Fig. 3a). The adults of the extant taxa exhibit an overlap in morphospace but can be separated to some extent along PC2. *U. arctos* occupies a wide range along PC2 so that the adult specimens overlap with the other species. Adult *U. maritimus* exhibit a relatively plane and narrow cranial shape compared to all other groups. The braincase is usually relatively flat and long and the internasal suture (Landmark 4) is shifted rostrally, leading to long nasal region (Fig. 3a). On the contrary, the crania of adult *U. spelaeus* as well as some juveniles of *U. spelaeus* and *U. arctos* have a relatively shorter and broader braincase with high vaulted calvaria. The nasals and palates are also relatively shorter and wider. An intermediate state can be observed in *U. arctos* and *U. americanus*. Unlike the first two principal components, PC3 does not distinguish between species or age stages (Additional file 2: Figure A1a).

Computing Procrustes distances between juveniles and adults of the analysed species revealed that the species are significantly different from one another (Table 3). Moreover, the juvenile specimens are significantly different from their adult conspecifics (Table 3).

**Table 3:** Cranial and mandibular Procrustes distances between age classes and species

		*U. americanus*	*U. arctos*	*U. maritimus*	*U. spelaeus*	*U. arctos*
		adult	adult	adult	adult	juvenile
*(a) Procrustes shape variation of the cranium*						
*U. arctos*	adult	**0.0391 (<.0001)**				
*U. maritimus*	adult	**0.0528 (<.0001)**	**0.0597 (<.0001)**			
*U. spelaeus*	adult	**0.0785 (<.0001)**	**0.0657 (<.0001)**	**0.086 (<.0001)**		
*U. arctos*	juvenile	**0.1068 (<.0001)**	**0.1051 (<.0001)**	**0.1328 (<.0001)**	**0.1451 (<.0001)**	
*U. spelaeus*	juvenile	**0.0779 (0.0106)**	0.0682 (0.0685)	**0.1037 (0.0003)**	**0.0777 (0.009)**	**0.091 (0.041)**
*(b) Nonallometric shape variation of the cranium*						
*U. arctos*	adult	**0.0415 (<.0001)**				
*U. maritimus*	adult	**0.0494 (<.0001)**	**0.0597 (<.0001)**			
*U. spelaeus*	adult	**0.0554 (<.0001)**	**0.0473 (<.0001)**	**0.078 (<.0001)**		
*U. arctos*	juvenile	**0.064 (<.0001)**	**0.0426 (<.0001)**	**0.0798(<.0001)**	**0.0271 (0.0001)**	
*U. spelaeus*	juvenile	**0.0789 (<.0001)**	**0.0734 (0.0249)**	**0.1015 (0.0001)**	0.0439 (0.0916)	0.0626 (0.2624)
*(a) Procrustes shape variation of the mandible*						
*U. arctos*	adult	**0.0301 (<.0001)**				
*U. maritimus*	adult	**0.0461 (<.0001)**	**0.0411 (<.0001)**			
*U. spelaeus*	adult	**0.0523 (<.0001)**	**0.0565 (<.0001)**	**0.0784 (<.0001)**		
*U. arctos*	juvenile	**0.1109 (<.0001)**	**0.1003 (<.0001)**	**0.1179 (<.0001)**	**0.1335 (<.0001)**	
*U. spelaeus*	juvenile	**0.1001 (0.0002)**	**0.0949 (<.0001)**	**0.1218 (<.0001)**	**0.1002 (<.0001)**	**0.0741 (0.0183)**
*(b) Nonallometric shape variation of the mandible*						
*U. arctos*	adult	**0.0417 (<.0001)**				
*U. maritimus*	adult	**0.0504 (<.0001)**	**0.0416 (<.0001)**			
*U. spelaeus*	adult	**0.0517 (<.0001)**	**0.0442 (<.0001)**	**0.0733 (<.0001)**		
*U. arctos*	juvenile	**0.0659 (<.0001)**	**0.0382 (<.0001)**	**0.0666 (<.0001)**	**0.0371 (0.0025)**	
*U. spelaeus*	juvenile	**0.081 (0.0002)**	**0.077 (<.0001)**	**0.1046 (<.0001)**	0.0418 (0.1602)	0.0685 (0.0849)

Procrustes distances for non-size corrected and size corrected variation is given. Significance values are given in brackets and significant results are in bold.

### Shape variation in mandibular morphology

The first three principal components of mandibular shape space account for 64.04 % of the total variation. The PCA of the mandibular landmarks presents a different distribution of the species and age classes in morphospace (Fig. 3b) than the cranial analysis (Fig. 3a). PC1 displays a slight gradient from juveniles (positive values) to adults (negative values), although overlap of the confidence ellipses is extensive (Fig. 3b). The mandibular shape modification from juvenile to adult is generally characterised by the relative enlargement of the coronoid process, the reduction of relative tooth row length (p4 to m3), and the change in the angle between the buccal and labial part of the mandible. PC2 separates adult *U. maritimus* (negative values) from *U. spelaeus* (positive values) (Fig. 3b). In contrast to *U. spelaeus*, *U. maritimus* features relatively large coronoid processes, long tooth rows (p4 to m3), and an enlarged anterior part of the mandible. *U. arctos* and *U. americanus* exhibit an intermediate shape (centred around 0). PC3 does not separate species or age stages (Additional file 2: Figure A1b).

The comparison of mandibular Procrustes distances across the species and age stages revealed similar results as in the cranium. The adults of the investigated species are significantly different from each other and the juveniles are different from their adult conspecifics (Table 3).

### Cranial and mandibular shape changes in relation to size across ontogenetic stages

The multivariate regressions of cranial and mandibular shape on size across all species revealed a significant correlation in both cases (*p* < 0.0001). The angular comparisons of the ontogenetic trajectories of *U. arctos* and *U. spelaeus* that are reflected in cranial and mandibular shape changes associated with size are more similar than chance (cranium, angle = 39.3°, *p* < 0.00001; mandible, angle = 51.77°, *p* = 0.0014).

After removing the between-species effect of size on cranial shape, PC1 separates adult *U. spelaeus* (negative PC1 values) from adult *U. maritimus* (negative PC1 values) (Additional file 3: Figure A2a). The characteristics which are not size-related are relatively expanded and massive zygomatic arches, a higher braincase, short nasals, a dorsally rotated rostrum, and a broad palate in *U. spelaeus* and narrow zygomatic arches, a flatter braincase, and an overall narrower skull in *U. maritimus* (Additional file 3: Figure A2a). Juveniles are no longer separated from their adult conspecifics considering the confidence ellipses alone (Additional file 3: Figure A2a). PC2 and PC3 do not separate species or age classes (Additional file 3: Figure A2a, b). Removing the effect of size on mandibular shape results in a lack of separation of species and age classes (Additional file 3: Figure A2c, d).

The visually observed lack of differentiation of species and age stages in the non-allometric cranial and mandibular shape space (Additional file 3: Figure A2) is only partially reflected in the Procrustes distances. Species can still be significantly distinguished from one another when considering adults, both in the cranial and mandibular shape space (Table 3). Differences between juveniles disappear in the non-allometric shape space (Table 3). The juvenile *U. arctos* are still significantly different from their adult conspecifics but juvenile *U. spelaeus* are not distinguishable from adult *U. spelaeus* (Table 3).

### Phylogenetic comparison

Permutation tests revealed that the null hypothesis of a lacking phylogenetic signal cannot be rejected: the p-values for cranial and mandibular data were non-significant, either when using branch length estimates from Krause et al. [16] (p_cranium_ = 0.67, p_mandible_ = 0.49) or branch lengths set equal to 1 (p_cranium_ = 0.66, p_mandible_ = 0.49).

## Discussion

We explored the distribution of extinct *U. spelaeus* (cave bear) and three extant bear species *U. maritimus* (polar bear), *U. arctos* (brown bear), and *U. americanus* (black bear) in morphospace, investigating cranial and mandibular shape changes in ontogeny of (*U. arctos* and *U. spelaeus*) and adult shape changes among all investigated species. The cranial and mandibular shapes in adults of the different species are significantly distinguished from the cranial and mandibular shapes in adults of the other species. Concerning the cranium, these changes are not only size related. Juvenile *U. arctos* and *U. spelaeus* are significantly distinguished from one another and from their adult conspecifics (Table 3) but these differences disappear when the effect of size is removed (Table 3, Additional file 3: Figure A2). Thus, cranial and mandibular shape in *U. arctos* and *U. spelaeus* are already different during the juvenile period and these differences are probably mostly size-related. There is a similar pattern of cranial growth in *U. spelaeus* and *U. arctos*, as shown by similarity of angles between the ontogenetic trajectories. The postnatal ontogenetic cranial and mandibular shape changes in *U. spelaeus* and *U. arctos* can generally be described as affecting especially the relative length, width, and height of the braincase, length and width of the rostrum, width of the zygomatic arches, and height of the coronoid process. These findings are consistent with quantitative descriptions of *U. americanus* [38]. Considering juvenile *U. spelaeus*, all these results have to be interpreted with caution because the sample size is restricted and the used tests might therefore fail to detect differences between species or age classes. Although species and ontogenetic stages are better differentiated in the cranial shape space than in the mandibular shape space (Fig. 3), both shape spaces exhibit ontogenetic modifications. In both structures, the species are significantly distinguishable and juveniles can be differentiated from their adult conspecifics (Table 3).

Significant differences of the cranial shapes between juveniles and adults in *U. spelaeus* and *U. arctos* could potentially result from dietary shifts in the course of the weaning period. We think that this is unlikely. The weaning period in *U. arctos* ranges from 18 to 30 months (Fig. 1) and thus roughly corresponds with the age at which the adult dentition is completed in our sample of *U. arctos* (from one year of age onwards, Additional file 1: Table A1). However, the cubs are already eating a variety of foods by about 5 months of age [49], an age at which the here examined *U. arctos* specimens do not yet have their complete adult dentition and are thus still categorised as juveniles (Additional file 1: Table A1). Similarly, the age at the attainment of sexual maturity in *U. arctos* is much later than the above reported age at completion of the adult dentition (Fig. 1). Therefore, neither the age at weaning, nor the age at attainment of sexual maturity appear to correlate with the observed cranial and mandibular shape changes between juveniles and adult in *U. arctos*. We thus infer that probably also the extinct *U. spelaeus* did not exhibit cranial and mandibular shape changes associated with these life history variables.

Differences in cranial shape that differentiate adult *U. spelaeus* from extant relatives are not dependent on phylogenetic relatedness and size (Fig. 3, Additional file 3: Figure A2, Table 3) but could be related to diet. Hereby, PC2 (PC1 in the non-allometric shapespace. Additional file 3: Figure A2a) might represent a gradient from carnivory to herbivory (Fig. 3, Additional file 3: Figure A2a), although overlap is partially extensive. Non-parametric Kruskal-Wallis tests and post-hoc comparisons (“pgirmess” package version 1.5.9 [50] implemented in R) confirmed a significant difference of values for PC2 among the three dietary categories (herbivorous, omnivorous, carnivorous) in the cranial landmarks (chi^2^ = 162.5, *p* < .00001) and in the mandibular landmarks (chi^2^ = 98.1, *p* < .00001) as well as significant differences among all three groups (*p* < 0.05). The carnivorous *U. maritimus* is characterised by a relatively long rostrum and flat braincase, whereas herbivorous *U. spelaeus* exhibit a relatively higher and wider braincase and a shorter rostrum. The omnivorous *U. americanus* occupies the same cranial and mandibular morphospace as the omnivorous *U. arctos* (Fig. 3). These findings are consistent with the results of Figueirido et al. [29], who investigated ecomorphologically correlated cranial and mandibular shape variation in ursines, finding shared craniodental traits, similar to ours, among herbivorous bear species and opposite features in carnivorous bears. Omnivorous bears showed intermediate craniodental morphology. However, since the allocation of *U. spelaeus* to an herbivorous diet has been debated [51, 52], the allocation of PC2 to diet is arguable.

Many previous studies on skull morphometrics restricted themselves to either the cranium or the mandible as markers of morphological change [10, 12, 27, 31–33]. This is a reasonable approach, but it is clear that both parts document different degrees of complexity and can reveal different patterns, even if correlated [11, 53]. Differences in the growth trajectories of the mandible and the cranium were found in this work, as had also been found in sabercats [54]. Independent inheritance of upper and lower jaw features have been reported in hybrids of different dog breeds [55]. Some descendants of such cross breeds inherit the upper jaw of one parent and the lower jaw from the other. These features indicate the possible independence in the ontogenetic development of mandible and cranium. In this regard it would be valuable and appropriate not to consider the cranium and the mandible separately, but to investigate both structures.

## Conclusions

The cranial and mandibular shape disparity in the *Ursus* lineage appears to be more influenced by adaptation to diet than by size or phylogeny. In contrast the cranial and mandibular shape changes during postnatal ontogeny in *U. arctos* and *U. spelaeus*, leading to species specific and feeding ecology related adult skull shape, are probably largely size related. Shape differences between juveniles and adults are probably not related to age at weaning or attainment of sexual maturity. As the skull is a more complex structure than the mandible, the study of the former is a richer source subject for studies of growth.

### Availability of supporting data

All the supporting data are included as additional files.

## Additional files

Additional file 1: Table A1. List of all examined crania and mandibles. ID, collection number; IDAS, individual dental age stage [37]; sex, male (m) and female (f); absolute age is given in days (d), weeks (w), months (mo), or years (y). (XLSX 33 kb) Additional file 2: Figure A1. Principal component analysis of cranial (A) and mandibular (B) shape variation in juvenile and adult bear species. Ellipses represent the 95 % confidence interval of the age stages (juvenile and adult) within species. (TIFF 568 kb) Additional file 3: Figure A2. Principal component analysis of the non-allometric component of cranial (A,B) and mandibular (C,D) shape variation in juvenile and adult bear species. Ellipses represent the 95 % confidence interval of the age stages (juvenile and adult) within species. Cranial and mandibular models represent extreme shapes on PC1 and PC3. (TIFF 1428 kb)

## Abbreviations

3D: Three-dimensional
d: Days
f: Female
GM: Geometric morphometric
IDAS: Individual dental age stage
IfPEN: Institut für Paläontologie Erlangen, Erlangen, Germany
IPUW: Institut für Paläontologie Wien, Vienna, Austria
m: Male
mo: Months
MCP: Château de Montbéliard, Montbéliard, France
NHMW: Naturhistorisches Museum Wien, Vienna, Austria
NMB: Naturhistorisches Museum Basel, Basel, Switzerland
NMBE: Naturhistorisches Museum Bern, Berne, Switzerland
NMSG: Naturmuseum St. Gallen, St. Gallen, Switzerland
PC: Principal component
PCA: Principal component analysis
RBIN: Institut Royal des Sciences Naturelles de Belgique, Brussels, Belgium
*U*: *Ursus*
w: Weeks
y: Years
ZMUZH: Zoologisches Museum der Universität Zürich, Zurich, Switzerland
