# Supplementary figures and images for: Growth trajectories in the cave bear and its extant relatives: an examination of ontogenetic patterns in phylogeny
Source: BMC Evol Biol. 2015 Nov 2;15:239. doi: 10.1186/s12862-015-0521-z (PMC4630841; doi:10.1186/s12862-015-0521-z)

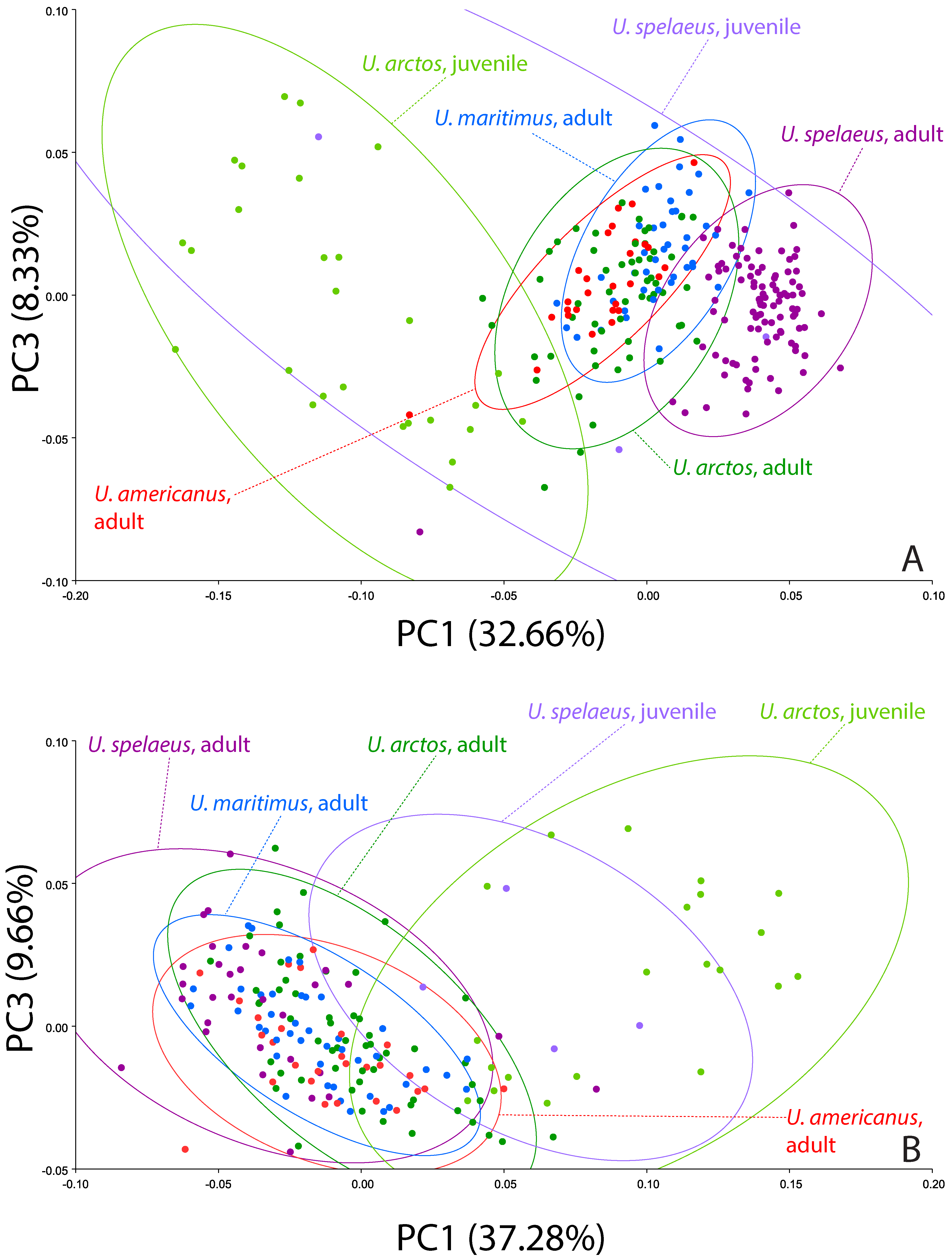

Supplement: Additional file 2: Figure A1. — Principal component analysis of cranial (A) and mandibular (B) shape variation in juvenile and adult bear species. Ellipses represent the 95 % confidence interval of the age stages (juvenile and adult) within species. (TIFF 568 kb) [file 12862_2015_521_MOESM2_ESM.tif]
